# Why Is the Baker Classification Inadequate for Classifying Silicone Implant Fibrous Capsules?

**DOI:** 10.7759/cureus.55776

**Published:** 2024-03-08

**Authors:** Eduardo De Faria Castro Fleury

**Affiliations:** 1 Radiology, MD duFLE Diagnósticos, São Paulo, BRA

**Keywords:** classification, breast implants, inflammation, silicones elastomers, silicones

## Abstract

Baker's clinical classification is adopted as the gold standard for grading complications related to silicone implants. Despite being widely used for this purpose, the classification has several limitations, highlighting subjectivity, reproducibility, and interobserver agreement. In this technical report, we aim to present the reasons for the inadequacy of the Baker classification for breast implants and the main factors contributing to false-negative results using recent theories of surface tension of fluids and gel bleeding. We also present an alternative classification proposal using magnetic resonance imaging of the breasts.

## Introduction

Baker's clinical classification is adopted as the gold standard for grading complications related to silicone implants and ranges from 1 to 4, where 4 indicates greatest severity [1]. Despite being widely used for this purpose, the classification has several limitations, highlighting subjectivity, reproducibility, and interobserver agreement [2].

The main objective of the classification is to diagnose and grade the most common complication related to implants: capsular contracture. The Baker classification is performed by a trained physician and supported by inspection findings and palpation of the breasts. As expected, changes in implants and fibrous capsules (FCs) are obtained indirectly [1,2].

As it is performed indirectly, it is impossible to assess the presence of gel bleeding, an event described as gel extravasation in intact implants and recognized by the United States Food and Drug Administration (FDA) as common in silicone implants [3].

In this technical report, we aim to present the main reasons for the inadequacy of the Baker classification for breast implants, discuss the main factors that contribute to false-negative results using recent theories of surface tension of fluids, and present an alternative classification proposal using magnetic resonance imaging (MRI) of the breasts.

## Technical report

FC and silicone implant

Silicone implants are medical devices classified in class III according to the FDA, which require general controls and premarket approval. According to the FDA's risk-based classification, Class III includes those with the most significant risk [4].

Silicone implants consist of an outer shell made of a more rigid elastomer and filled with a component that can be liquid (saline), silicone gel, or mixed (double-lumen). The elastomer and the silicone gel are composed of polydimethylsiloxane in shorter or longer chains, depending on the purpose. The surface of the elastomers can vary between smooth, macro-textured, and microtextured; in some cases, it can have an external polyurethane component for better adherence to breast tissue. The latest generations of silicone implants use highly cohesive gel for filling. The purpose of the cohesive gel, and the surface type association allow better adaptation of the silicone to the surgical site, reducing the possibility of gel extravasation and capsular contracture [5,6].

Briefly and outside the main scope of this theoretical article, after silicone implant placement, the human body evokes an inflammatory response that results in FC formation on the periphery of the foreign body. Over time, the FC is expected to thicken due to chronic inflammation. The FC thickening reduces the pocket volume created after the implant placement. Also, it reduces the FC's elastic capacity due to fibroblasts' presence [7,8]. Theoretically, the implants must maintain their volume, and reducing their space will result in contracture. Generally, capsular contracture is expected to appear ten years after implant placement, which is the FDA-recommended period for implant replacement [3].

Surface tension and elastic capsules

The physical deformation of elastic capsules in general in the face of impacts is virtually unexplored by current science and was the subject of publication in 2020 by Jambon-Puillet et al. [9]. Capsules combine liquid and solid phases. In the article, the authors compared capsules with drops of water. Upon impact, the deformation of a drop indeed results from a delicate balance between inertia, viscous effects, and capillarity. Unlike drops, however, the presence of a shell that bends and stretches to contain the liquid prevents the formation of singular structures and the fragmentation into multiple droplets [9]. 

Jambon-Puillet et al. observed that thinner capsules deformed more than thicker and larger ones in the experiments. Capsules are often closed shells and thus must stretch to bend. The elastic and isotropic elastomers can be described by a strain energy density (per unit undeformed volume). They assumed the capsule to be initially spherical and that the deformation during impact remains axisymmetric [9].

According to internal content viscosity, higher viscosity fluids result in a lower maximal extension of the elastomer. This effect is nonlinear: a 10-fold increase in viscosity between silicone oil and glycerol has a similar effect as the 1,000-fold increase between glycerol and water. The relationship between the deformity of the elastomer and the amount of internal content must be "d=0" for models with the capsule configuration in the original, undeformed state, which keeps the tension on the elastomer stable [9].

Suppose the elastomers are impermeable and the surface is intact, with constant internal content. In that case, the increase in the FC thickness around the implant's shell will determine an increase in surface tension in the silicone implants, which results in contracture. In these cases, an increase in the anteroposterior diameter of the spheres is observed, from oblate spheroid to prolate spheroid.

However, if the filling of the spheres is less than their internal capacity, there will be no tension on the elastomer shell in the condition of FC thickening.

Baker classification

**Figure 1:**
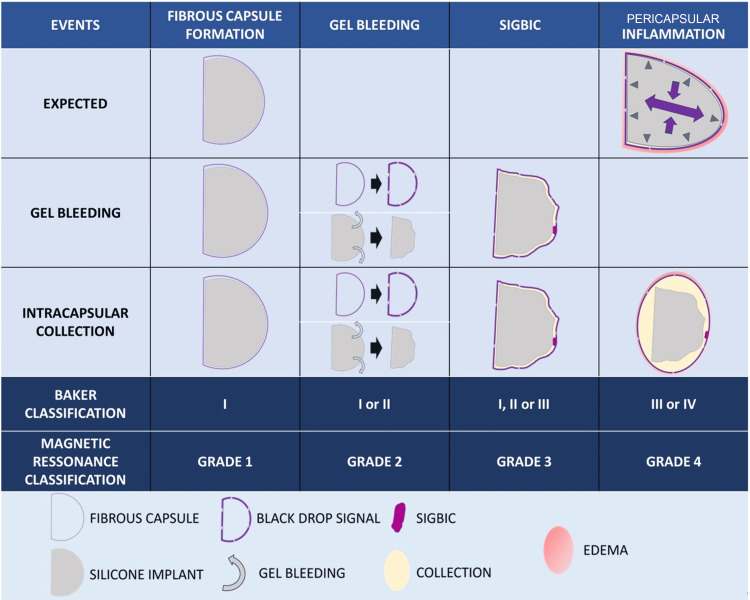
Evolutionary complications of silicone implants and fibrous capsules, correlated with Baker's classification and the proposed imaging classification SIGBIC: silicone-induced granuloma of silicone implant capsule Image Credit: Eduardo de Faria Castro Fleury

By observing the clinical evolution of patients with silicone implants and adopting the concepts described in the relationship between the FC and the silicone implant, Baker, in 1978, proposed classifying silicone implants into four evolutionary scales, distributed as follows: Baker grade I, breast is normally soft and looks natural; Baker grade II, breast is a little firm but looks normal; Baker grade III, breast is firm and looks abnormal; and Baker grade IV, breast is hard, painful, and looks abnormal (Figure 1) [1].

In an article published in 2020 by de Bakker et al., the authors discuss why the Baker classification for capsular contracture is unreliable as a diagnostic tool. They conclude that interobserver feasibility and observer agreement could have been better and advocate for a new classification to classify late complications related to implants [2].

Gel bleeding

In 2020, the FDA recognized that silicone implants show surface degradation over time, and the internal contents leak through the shell even when intact [3]. According to FDA Breast Implant Guidance, “The patient should also be informed that most of these chemicals stay inside the shell of the implant but small quantities have been found to diffuse (gel bleed) through the implant shell of silicone gel-filled implants, even if the implant is intact and not ruptured or leaking” [3]. This phenomenon was confirmed by Djikman et al., who observed free silicone particles in the FC of the implants [10]. They also demonstrate evidence regarding the permeability of FCs, where they observed silicone particles in the pericapsular space.

However, there may still be a third variable in this equation involving the FC and silicone implant, which would be the presence of a third element: intracapsular collection. The inflammatory response of the FC to the silicone implant can produce exudate in variable amounts depending on the intensity of the inflammation [5,8]. When the amount of exudate produced exceeds the amount of extravasated gel, tension will increase within the FC, determining the deformation of the capsule's morphology. These cases are generally classified into Baker categories III and IV.

Magnetic resonance classification

MRI is the gold standard imaging method for evaluating silicone implants. The latest edition of the Breast Imaging Reporting and Data System (BI-RADS) lexicon has a dedicated section for implants; however, the lexicon does not present descriptors to classify changes in the FC that allow diagnosing and classifying contractures. Complementary silicone-sensitive sequences added to the conventional protocol for studying breasts allow for a better assessment of changes [11].

Recently, de Faria Castro Fleury and Castro described the presence of silicone-induced granuloma inside FCs as silicone-induced granuloma of silicone implant capsules (SIGBIC) and proposed a dedicated classification to evaluate silicone implants [12]. SIGBIC would be the primary imaging marker of silicone extravasation in intact breast implants and an indicator of involvement of the FC by acute and chronic inflammatory processes. In clinical observation, we observed that episodes of inflammation of the FCs are remitting and recurrent [8].

In 2020, de Faria Castro Fleury published an article demonstrating that SIGBIC was present in 30.5% of patients who underwent MRI examinations in our service, with an average onset time of seven years [13]. In the article, they demonstrated that the main descriptors in the BI-RADS lexicon associated with SIGBIC were water droplet, enlarged intramammary lymph node, pericapsular edema, and intracapsular seroma. Water droplet is the only describer present in the current BI-RADS lexicon that could be associated with changes in the permeability of the implant shell and gel extravasation.

The diagnosis of SIGBIC uses three unequivocal criteria for diagnosis: black drop signal, mass with hyper signal at T2 weighted sequence, and late contrast enhancement. The black drop signal is a marked focus of low signal at T1 sequences in the FC, and a silicone signal focus could be associated with silicone-sensitive sequences without enhancement in post-contrast sequences. Mass with the hyper signal at T2 weighted sequence corresponds to an intracapsular mass that could be misdiagnosed as a seroma. Late contrast enhancements are delayed contrast enhancement of the mass, indicating low vascularization [12].

The MRI classification proposed to evaluate FC consists of four grades. The grades vary according to the impairment degree of the FC and are evolutionary, starting from the mildest findings (Grade 1) to the most compromised (Grade 4). To classify FC, three MRI sequences are required: axial T2* weighted with fat suppression, sequences without enhancement in T1 dynamic weighted sequences after contrast administration, and sagittal with proton density. Maximum intensity projection (MIP) reconstructions are used to evaluate FC enhancement and the presence of pericapsular edema. In addition to the FC sequences, the MRI protocol includes silicone-sensitive sequences to evaluate silicone implant contents.

**Figure 2:**
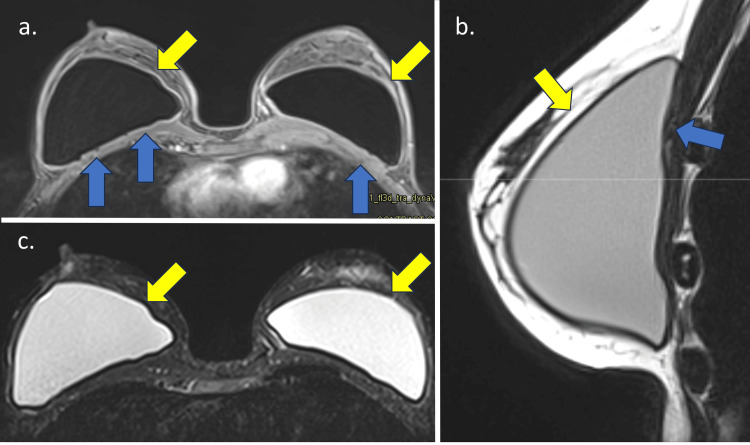
Breast MRI sequences showing MRI fibrous capsule Baker classification Grade 2 in a 42-year-old patient with silicone implants for four years. The Baker classification was Grade 2. (a) Axial T1 post-contrast sequence shows fibrous capsular thickening with contrast enhancement (yellow arrows) associated with black-drop signal (blue arrows); (b) The sagittal proton-density sequence shows the same findings; (c) Axial STIR sequence confirms the capsular thickening. STIR: short tau inversion recovery Image Credit: Eduardo de Faria Castro Fleury

**Figure 3:**
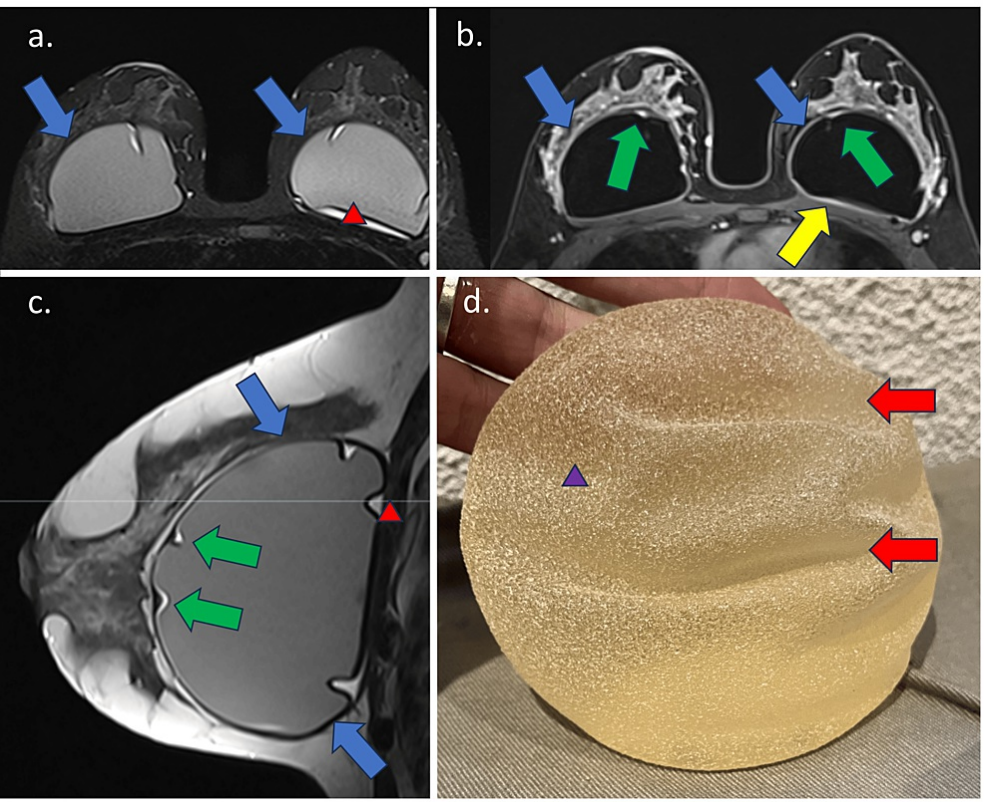
Breast MRI sequences showing MRI fibrous capsule classification Grade 3 in a 39-year-old patient with silicone implants for six years. The Baker classification was Grade 2. (a) Axial STIR sequence shows fibrous capsule thickening, especially in the left breast (blue arrow), associated with intracapsular collection (red triangle); (b) T1 post-contrast sequence shows fibrous capsular thickening (blue arrow) with intracapsular focal thickening of the fibrous capsule associated with late contrast enhancement (green arrow); (c) The sagittal proton-density sequence shows the same findings. The axial STIR sequence confirms the capsular thickening; (d) The macroscopy of the implant shows degradation of the shell surface (purple triangle) with volume loss determining folds in the shell surface (red arrow). STIR: short tau inversion recovery Image Credit: Eduardo de Faria Castro Fleury

**Figure 4:**
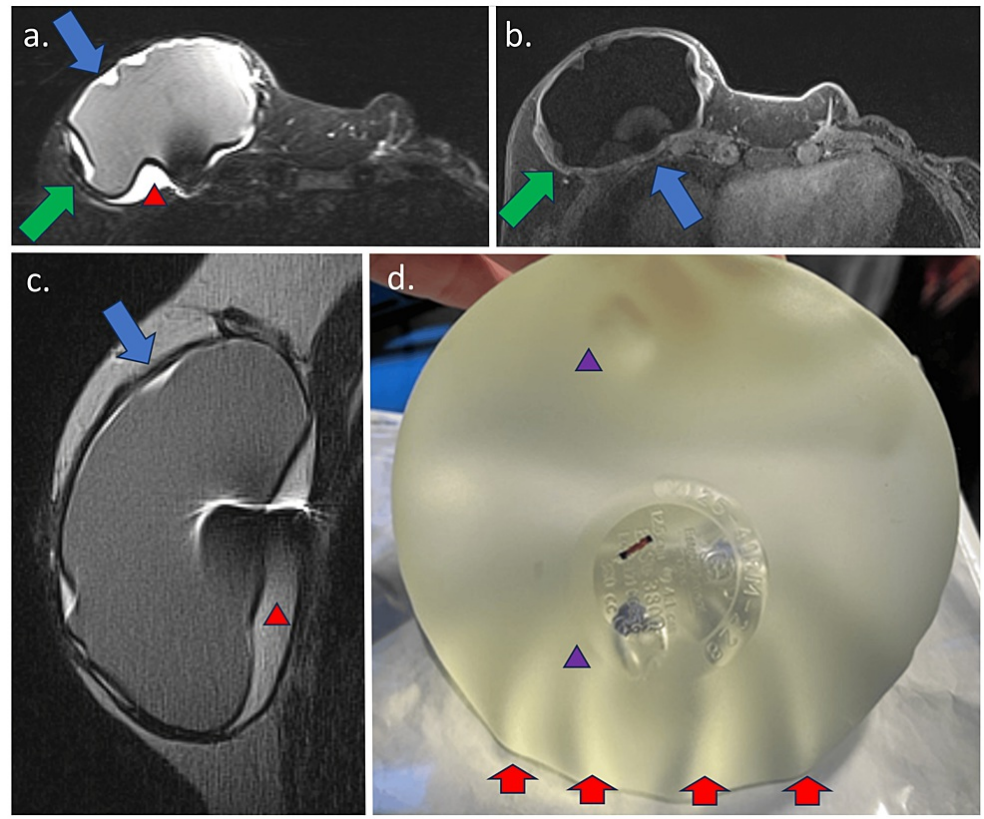
Breast MRI sequences showing MRI fibrous capsule classification Grade 4 and the macroscopy of the silicone implant in a 61-year-old patient with silicone implants for four years. The Baker classification was Grade 2. (a) Axial STIR sequence shows fibrous capsule thickening (blue arrow) associated with intracapsular mass (green arrow) and associated with intracapsular collection (red triangle); (b) T1 post-contrast sequence shows fibrous capsular thickening with a black-drop signal (blue arrow) with intracapsular focal thickening of the fibrous capsule associated with late contrast enhancement (green arrow); (c) The sagittal proton-density sequence shows the same findings associated with moderate intracapsular collection; (d) The implant's macroscopy shows permeability loss as droplets inside the silicone implants (purple triangle), with volume loss determining folds in the shell surface (red arrow). STIR: short tau inversion recovery Image Credit: Eduardo de Faria Castro Fleury

In grade 1, FC shows no detectable changes, mild wall thickening, mild contrast enhancement, and increased anteroposterior diameter. The MIP reconstruction is negative. Grade 2 accentuates the FC wall thickening and enhancement (less than 5.0 mm) with the appearance of a black-drop signal. The MIP reconstruction is negative (Figure 2). In Grade 3, in addition to Grade 2 findings, a focal thickening (more than 5.0 mm) of the FC or an intracapsular heterogeneous tissue with late contrast enhancement (silicone-induced granuloma) is observed. The MIP reconstruction is negative (Figure 3). Finally, Grade 4 shows significant FC thickening and enhancement, with pericapsular enhancement. It may be associated with the discontinuity of the FC. The findings denote an inflammatory process, most often associated with a high pericapsular signal in short tau inversion recovery (STIR) sequences. The MIP reconstruction is positive (Figure 4) [12].

In a 2022 study, Fleury and Castro determined the interobserver variation using the proposed classifier, with interobserver agreement of k=0.65, substantial agreement, in the first test round [12].

## Discussion

In 2020, the FDA revised the recommendations regarding silicone implants. The new recommendations include screening patients with asymptomatic implants five years after implantation surgery, followed by follow-up every two years. The FDA recommends screening with MRI. Another important point highlighted by the FDA was considering that implants are not lifelong devices and that they have an average lifetime of 10 years. Finally, another relevant point was recognizing gel bleeding to be real [3,4].

Since 1978, Baker's clinical classification has been used to classify capsular contractures in breast implants. Despite being simple, the classification is subjective and indirect, which makes its reproducibility difficult and favors low interobserver agreement [1,2]. The main variables for Baker's classification are FC thickness and silicone implant volume. For the Baker classification to be valid, the volume of the silicone implant must be constant, while the thickness of the FC varies progressively.

The article published by de Bakker et al. appears to be the most current article on the impact of the Baker classification for breast implants [2]. They demonstrate that the interobserver reliability could have been better, as was the interobserver agreement, which is inconsistently defined in reference works and scientific literature. They also discussed that an alternative to the Baker classification could be applanation tonometry, described as unreliable in clinical practice [2]. In the Netherlands, the reimbursement by health insurers for surgical treatment of FC complications relies on a combination of the Baker classification grade IV with reported local pain, according to the article, which would be the main impact on patient care of a failed classification [2]. 

When considering gel bleeding reducing the silicone implant volume, and if the volumetric loss of the sphere is more significant than the thickening of the FC, the Baker classification will fail to determine the impairment of the FC. It is expected that silicone implant surfaces and the cohesive gel filling prevent the formation of singular structures and the fragmentation into multiple droplets [9]. However, transforming the initial homogeneous content into multiple droplets favors bleeding through the semipermeable capsules. Some studies demonstrate that extravasated silicone can migrate to different body parts, from the pericapsular region to distant organs [10].

Until recently, silicone extravasation was believed to be rare in new generations of implants, and extravasated particles were inert to the human body [14]. This concept has already been changed, supported by the latest recommendations from the FDA, which recognizes gel bleeding and relates it to some clinical symptoms and diseases of the immune system [3,4]. When the Baker classification is used, patients with gel bleeding may have a tragic outcome because, despite the clinical symptoms present, the clinical diagnosis of silicone changes is negative. The false negative result delays the diagnosis of complications related to implants and impacts the management and follow-up of these patients. Many patients with clinical symptoms related to silicone end up being treated for psychiatric disorders [15-17].

In this context, MRI has the potential to classify implants due to the possibility of directly visualizing the implants, the FC, and the intracapsular content [5,7,8]. The appropriate use of magnetic resonance provides additional information regarding gel bleeding and grades FC impairment [12,13].

As the FDA already recommends MRI for silicone implant screening, the impact on patient care would be to provide objective data on the compromise of breast implants in changes that are neglected by the Baker classification [4,12].

Since the beginning of the 2000s, silicone implants have been evaluated by MRI according to the BI-RADS lexicon proposed by the American College of Radiology (ACR), universally accepted in clinical practice [11]. However, the BI-RADS lexicon presents descriptors for silicone implants. The lexicon does not have a dedicated section to describe and evaluate FCs. In researching the available literature, there appears to be a lack of studies dedicated to assessing and classifying breast silicone implant FCs using imaging methods. The classification proposed by the author aims to fill the imaging diagnostic gap of FC complications and to promote a discussion of alternatives to the Baker classification in the scientific community.

## Conclusions

The Baker classification for evaluating silicone implants is limited when considering gel bleeding. Currently, the Baker false negative result directly impacts the diagnosis, management, and follow-up of patients suffering from silicone implant complications. Due to direct and objective assessment, MRI could be an alternative to classify FCs and silicone breast implants.
